# Circadian rhythm disruption impairs time-dependent dentine regeneration and reprograms the late-stage dental proteome in a mouse pulp injury model

**DOI:** 10.3389/fphar.2026.1770288

**Published:** 2026-06-17

**Authors:** Melike Guvendi, Hayriye E. Yelkenci, Elif Ozbay, Halil I. Koc, Olgu E. Tok, Serdar Altunay, Ertugrul Kilic, Mustafa Gundogar, Mustafa C. Beker

**Affiliations:** 1 Department of Endodontics, Faculty of Dentistry, İstanbul Medipol University, İstanbul, Türkiye; 2 Regenerative and Restorative Medical Research Center (REMER), Research Institute for Health Sciences and Technologies (SABITA), Istanbul Medipol University, Istanbul, Türkiye; 3 Department of Histology and Embryology, School of Medicine, Istanbul Medipol University, Istanbul, Türkiye; 4 Department of Physiology, School of Medicine, Istanbul Medipol University, Istanbul, Türkiye; 5 Department of Physiology, School of Medicine, Istanbul Medeniyet University, Istanbul, Türkiye

**Keywords:** dental pulp injury, dentine regeneration, dentinogenesis, proteomics, regenerative endodontic procedures, vital pulp therapy

## Abstract

Circadian rhythms coordinate metabolic and regenerative programs, yet their contribution to pulp healing and tertiary dentinogenesis remains unclear. Here, we investigated whether chronic circadian rhythm disruption (CRD) alters time-dependent dentine repair and proteomic remodeling after dental pulp injury (DPI) in male BALB/c mice. Animals were maintained either under a stable 12:12 h light-dark cycle or under a chronic jet-lag model with a daily 2 h phase advance. DPI was induced in the maxillary first molars, followed by immediate direct pulp capping with mineral trioxide aggregate and sealing with glass ionomer cement. Histological and histomorphometric analyses were performed at days 7, 28, and 56 post-injury, while open-field and light/dark transition tests were conducted on day 56. Untargeted LC-MS/MS proteomics was additionally performed on day-56 dental tissues to define injury-, circadian-, and dual-regulated molecular signatures. CRD produced a behavioral phenotype characterized by reduced locomotor activity and increased anxiety-like behavior. Histology revealed a canonical reparative pattern after DPI, with peripheral tertiary dentine formation surrounding a central connective tissue core; however, CRD consistently attenuated newly formed dentine area at all time points, indicating compromised odontoblast-like differentiation and mineral deposition. Proteomic profiling identified extensive remodeling of the dental proteome, with injury- and CRD-dependent signatures converging on metabolic regulation, cytoskeletal dynamics, immune pathways, and extracellular matrix organization. Integrated analyses delineated 204 injury-specific proteins, 128 CRD-specific proteins, and 86 co-regulated proteins, resolving the response into injury-dominant, circadian-dominant, dual-regulated, and condition-independent modules. Collectively, these results demonstrate that chronic circadian misalignment impairs dentine regenerative capacity and reprograms the dental proteome, suggesting that circadian status may be an underappreciated determinant of outcomes in vital pulp therapy and regenerative endodontic procedures.

## Introduction

Circadian rhythm is a fundamental regulatory system that orchestrates a wide range of physiological and pathophysiological processes across the 24-h day-night cycle. Through the coordinated action of hormonal, metabolic, and neuronal signals, central and peripheral clocks synchronize cellular activities to maintain temporal homeostasis (Mendoza, 2025). External cues such as light, temperature, and feeding cycles modulate these oscillators, ensuring proper alignment between environmental conditions and the internal biological clock (Reinke and Asher, 2019; Patke et al., 2020). Disruption of this delicate rhythmicity impairs tissue repair, immune responses, metabolic pathways, and neurophysiological functions.

Research on circadian regulation in dental tissues has historically focused on tooth development and amelogenesis; however, emerging evidence suggests that the dental pulp also harbors functional molecular clocks with potential implications for tissue regeneration (Janjic and Agis, 2019). Increasing evidence indicates that circadian rhythms play an important role in tooth development and dentinogenesis (Papakyrikos et al., 2020). Odontoblasts, the specialized cells responsible for dentine formation, exhibit circadian patterns of collagen synthesis and secretion, with collagen production reported to be substantially greater during the daytime than at night. This rhythmic odontoblastic activity is thought to contribute to the formation of incremental growth lines in dentine (Papakyrikos et al., 2020). Supporting this concept, experimental lesioning of the suprachiasmatic nucleus, the central circadian pacemaker, abolishes rhythmic dentinal growth lines in rats, indicating a direct influence of circadian regulation on dentine apposition (Ohtsuka-Isoya et al., 2001). In addition, odontoblasts are target cells for circadian-regulated hormones such as melatonin, suggesting that dentinogenesis may also be modulated through hormonally mediated circadian pathways (Tao et al., 2016).

The dental pulp responds to caries, mechanical exposure, or trauma by initiating a regenerative cascade that leads to the formation of tertiary dentine, a protective mineralized layer designed to preserve pulp vitality (Morotomi et al., 2019). These reparative processes form the biological basis of vital pulp therapy (VPT), which aims to preserve pulp vitality and support dentine repair following pulp exposure (Duncan et al., 2019; Coll et al., 2025a; Coll et al., 2025b). Direct pulp capping represents a core VPT procedure designed to activate reparative dentinogenesis after pulp exposure. Calcium hydroxide remains widely used in many settings due to its long-standing clinical history and ability to induce hard tissue barrier formation (Iwamoto et al., 2006; Brizuela et al., 2017). Nevertheless, limitations such as material dissolution and variable barrier quality have encouraged the development and clinical adoption of calcium silicate-based biomaterials. Among these, mineral trioxide aggregate (MTA) has gained increasing attention for its favorable biological properties, including support of odontogenic differentiation and more predictable tertiary dentine formation (Li et al., 2015; Brizuela et al., 2017; Parirokh et al., 2018; Kosin et al., 2025). Recent studies have demonstrated favorable biological outcomes of contemporary VPT approaches using bioactive materials (Duncan et al., 2019; Coll et al., 2025b). However, despite these advances, the contribution of systemic biological factors-particularly circadian regulation-to the predictability of reparative dentinogenesis remains largely unexplored.

Animal models provide essential platforms for investigating the molecular mechanisms underlying pulp healing and dentine formation, as they enable *in vivo* evaluation of vascular, neural, and immune interactions that cannot be fully recapitulated *in vitro* (Nakashima et al., 2019; Li et al., 2024). Controlled experimental injuries in mice allow reproducible assessment of repair processes at cellular and molecular levels and provide an opportunity to examine systemic modifiers-such as circadian misalignment-that may alter regenerative competence (Nakashima et al., 2019). These models also provide an opportunity to investigate systemic modifiers, such as circadian disruption, that may influence reparative dentinogenesis (Nakashima et al., 2019).

Therefore, the aim of the present study was to investigate the impact of chronic circadian rhythm disruption (CRD) on dental pulp regeneration following dental pulp injury (DPI) in male BALB/c mice. We quantified connective tissue formation and tertiary dentine deposition under normal and disrupted circadian conditions and performed comprehensive proteomic profiling using liquid chromatography-tandem mass spectrometry (LC-MS/MS) to identify injury-, circadian-, and dual-regulated molecular signatures. We hypothesized that chronic disruption of circadian rhythmicity impairs pulp healing and dentine regeneration by altering regenerative signaling pathways and the proteomic landscape of dental tissues. To our knowledge, this is the first study to directly examine the interplay between DPI-induced dentine regeneration and CRD, providing novel insights into how systemic chronobiological disturbances may reshape the molecular landscape of dental tissue repair and potentially influence outcomes in VPT and regenerative endodontic procedures.

## Materials and methods

### Ethics statement

The study was conducted in accordance with the National Institutes of Health (NIH) guidelines for the care and use of laboratory animals and was approved by the Animal Research Ethics Committee of Istanbul Medipol University (approval number: E-38828770-604.01-4640). DPI and capping procedures were performed by a trained researcher (Melike Guvendi and Serdar Altunay), blinded to the circadian condition of the animals through coded group allocation by an independent researcher. Prior to the study, standardized pilot procedures were used to ensure consistency in injury depth and capping technique. Animals were housed under a constant 12-h light/dark cycle (lights on at 07:00 daily) with *ad libitum* access to food and water. Data acquisition and analysis were performed in a blinded manner with respect to experimental group assignment.

### Experimental groups

**FIGURE 1:**
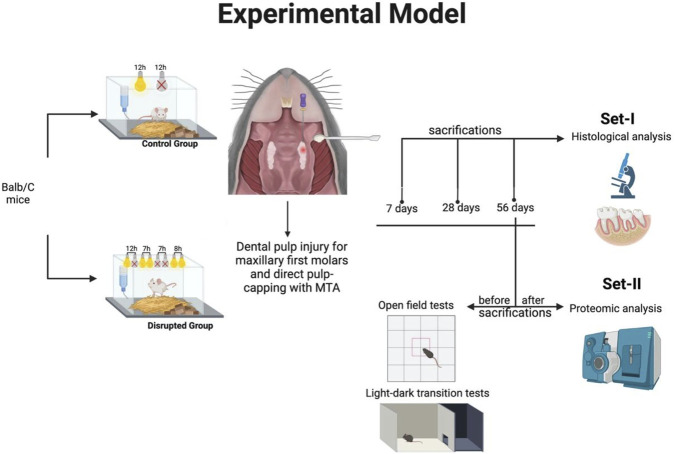
Schematic overview of experimental design. Male BALB/c mice were subjected to dental pulp injury (DPI) under either normal circadian conditions or chronic circadian rhythm disruption (CRD) induced by a jetlag protocol. Histological, behavioral, and proteomic analyses were performed at defined post-injury time points. Sham-operated control groups were included to assess circadian-dependent proteomic changes independent of injury. (Created with BioRender.com).

To evaluate the effect of CRD on dentine injury and repair, a total of 64 male BALB/c mice (8–12 weeks old) were used in this study (Figure 1). Fifty-six mice underwent DPI and were randomly assigned to two experimental groups: DPI-Control (DPI under a normal circadian rhythm; 12:12 h light-dark cycle; n = 28) and DPI-CRD (DPI under circadian rhythm disruption; n = 28). CRD was induced using a chronic jetlag protocol in which the light/dark cycle was advanced by 2 h per day, resulting in persistent circadian misalignment while maintaining the total duration of daily light exposure (Lawther et al., 2022). For histological assessment of dentine and connective tissue regeneration, mice from each DPI group were sacrificed at days 7 and 28 after injury (n = 8 per group at each time point). At day 56, the remaining animals (n = 12 per group) underwent behavioral testing, including the open field and light/dark transition tests, immediately prior to sacrifice.

Dental tissues obtained at day 56 from a subset of DPI animals were used for proteomic analysis, with four mice from the DPI-Control group and four mice from the DPI-CRD group included for LC-MS/MS based profiling. In addition, to investigate the impact of CRD on the dentine proteome under physiological, non-injured conditions, eight mice were assigned to sham groups: Sham-Control (no DPI, normal circadian rhythm; n = 4) and Sham-CRD (no DPI, CRD; n = 4). These animals were processed in parallel and sacrificed at day 56, and their dental tissues were included in the proteomic analyses to assess circadian-dependent effects independent of injury. All tissue collections were performed during the light phase to minimize circadian time of day dependent variability.

### Dental pulp injury model

Mice were anesthetized by intraperitoneal injection of ketamine (80 mg/kg) and xylazine (7.5 mg/kg). Body temperature was continuously monitored using a rectal probe and maintained at 36.5 °C–37.0 °C with a feedback-controlled heating system (69,020, RWD Life Science Co., Ltd., Shenzhen, China). Following confirmation of adequate anesthesia, animals were positioned in a supine posture, and the oral cavity was stabilized using a dental mouth holder to ensure reproducible access to the maxillary molars. DPI was performed on the maxillary first molars under an operating microscope (Zeiss OPMI Pico, Carl Zeiss, Jena, Germany). Occlusal cavities were prepared using a high-speed air turbine equipped with a 0.5 mm tungsten carbide bur under continuous water irrigation to minimize thermal damage. Cavity preparation was carefully controlled to achieve standardized pulp exposure without excessive dentin removal. Following visual confirmation of pulp exposure, mechanical injury to the pulp tissue was induced using a sterile stainless-steel K-file (#10; Dentsply Sirona, Charlotte, NC, United States), applied with gentle and consistent manipulation to ensure uniform pulp impairment across all specimens. After pulp injury, the exposed pulp tissue was immediately capped with mineral trioxide aggregate (MTA; ANPPK, Avalon Biomed, Bradenton, FL, United States), and the cavity was subsequently sealed with glass ionomer cement (Ketac™, Cat. No. 56633; 3M Oral Care, Germany) to prevent bacterial contamination and microleakage. Animals were then allowed to recover from anesthesia and returned to their home cages, where they were monitored daily for general health status and signs of distress.

At the predetermined experimental time points, mice were deeply anesthetized by intraperitoneal injection of ketamine (120 mg/kg) and xylazine (10 mg/kg). Adequate depth of anesthesia was confirmed by the absence of corneal and pedal withdrawal reflexes. Animals were euthanized by decapitation in accordance with institutional and international animal welfare guidelines, and tissue collection was initiated immediately following confirmation of death.

### Histological and histomorphometric analysis

Posterior maxilla of mice was fixed in 10% neutral buffered formalin (HT501128, Sigma-Aldrich, St. Louis, MO, United States) for 72 h at 4 °C. After fixation, the samples were rinsed with dH_2_O and decalcified in 14% ethylenediamine tetra-acetic acid (EDTA; E8008, Sigma-Aldrich, St. Louis, MO, United States) for 6 weeks at 4 °C. Then, the samples were rinsed with dH_2_O and gradually dehydrated by an ethanol series (70%, 90%, 96%, and 100%) before being cleared by xylene (564,056, Sigma-Aldrich, St. Louis, MO, United States). After that, the samples were submerged in liquid paraffin overnight at 60 °C and embedded in paraffin. From the paraffin blocks containing the first molar teeth of the maxilla, 5-μm-thick sections were obtained with a microtome (RM2265, Leica, Wetzlar, Germany) and placed on positively charged slides. The paraffin sections were stained with hematoxylin and eosin (H&E) and examined under a brightfield microscope (Axio Zoom.V16, Carl Zeiss, Jena, Germany). To identify the lesion center, serial sections were collected at 20 μm intervals; three consecutive sections from the lesion center were used for histomorphometry. Newly formed dentine and connective tissue areas were quantified using ImageJ (NIH, Bethesda, MD, United States). The mean of three sections per animal was used for statistical analyses.

### Open-field and light-dark transition tests

On day 56, mice underwent open-field and light/dark transition tests prior to tissue collection (Beker et al., 2022). The open field is a round arena (100 cm diameter), which is covered by a white plastic floor. A 35-cm-high side wall made of white polypropylene surrounds the platform. It allows the evaluation of spontaneous locomotor activity and exploration behavior. Each mouse was released near the wall and allowed to explore for 10 min freely. Mobility time and traveled distance in the maze, and time spent in the wall zone were tracked using Anymaze software (Version 4.99; Stoelting Co., Wood Dale, IL, United States). The light/dark transition test (40 cm × 20 cm x 20 cm) consists of two equal light and dark chambers separated by a divider with a 4 cm × 4 cm opening at the floor level. Each mouse was placed in the corner of the light chamber at distance from the dark chamber and monitored for 10 min. Animal paths were tracked with an imaging system (Anymaze Version 4.99, Stoelting Co., Wood Dale, IL, United States). The time spent in the light/dark section was evaluated and presented.

### Sample preparation for liquid chromatography tandem-mass spectrometry (LC-MS/MS)

Immediately after mice were sacrificed, molar teeth were isolated, frozen in liquid nitrogen, broken into small pieces, and homogenized in a solution containing 50 mM ammonium bicarbonate and a protease inhibitor cocktail (at a ratio of 1:100, 78,429, Thermo Fisher Scientific, Waltham, MA, United States). The homogenized samples were subsequently lysed at 95 °C using a protein extraction reagent kit (UPX Universal; Expedon, Heidelberg, Germany). After the lysis step, the samples were incubated for 1 hour at 4 °C, following the manufacturer’s guidelines. A Qubit 3.0 Fluorometer (Q33216, Invitrogen, Life Technologies) was utilized to determine protein concentrations. Once the homogenization and protein quantification steps were completed, the FASP (Filter Aided Sample Preparation) Protein Digestion Kit (ab270519, Abcam, Cambridge, United Kingdom) was employed to generate tryptic peptides following the manufacturer’s instructions. Specifically, 50 μg protein samples were filtered using a 30-kDa cutoff spin column with a 6 M urea solution. Subsequently, the samples were alkylated in the dark for 20 min at room temperature with 10 mM iodoacetamide. The next step involved an overnight incubation with MS grade trypsin protease (at a ratio of 1:100, 90,057, Thermo Fisher Scientific, Waltham, MA, United States) at 37 °C. The following day, peptides were eluted from the columns and subjected to lyophilization. After the lyophilization process, the peptides were reconstituted in a 0.1% formic acid solution (1,002,642,510, Merck KGaA, Darmstadt, Germany) and diluted to a concentration of 100 ng/μL before being injected into the LC-MS/MS system, which consisted of an ACQUITY UPLC M-Class coupled to a SYNAPT G2-Si high-definition mass spectrometer (Waters, MA, United States).

### LC-MS/MS analysis and data processing

LC-MS/MS and protein identification were performed with minor modifications according to previously published protocols (Beker et al., 2018; Beker et al., 2023). Briefly, samples were loaded onto the ACQUITY UPLC M-Class coupled to a SYNAPT G2-Si high-definition mass spectrometer (Waters, MA, United States). To equilibrate the columns, 97% of mobile phase (0.1% formic acid in LC-MS grade water) was used, and the column temperature was set to 55^o^C. Ninety-minute gradient elution from the trap column ACQUITY UPLC M-Class Symmetry C18 trap column (180 μm × 20 mm; 186,007,496, Waters, MA, United States) to the analytic column (ACQUITY UPLC M-Class HSS T3 Column, 100Å, 1.8 µm, 75 μm × 250 mm, 186,007,474, Waters, MA, United States) at 0.400 μL/min flow rate with a gradient from 4% to 40% hypergrade acetonitrile (100,029, Merck KGaA, Darmstadt, Germany) containing 0.1% formic acid (v/v) was used for the peptide separation. Positive ion modes of MS and MS/MS scans with 0.6-s cycle time were performed sequentially. All the ions within 50–1900 m/z range were fragmented in resolution mode without any precursor ion preselection. 100 fmol/μL Glu-1-fibrinopeptide B was used for lockmass reference with a 60-s interval to observe the mass stability.

Data were performed in Progenesis-QI for proteomics software (Waters, MA, United States) to identify and quantify peptides. At least one unique peptide sequence identified whole proteins and then the expression ratio of proteins was calculated. The pathway analysis was conducted using The Database for Annotation, Visualization, and Integrated Discovery (DAVID; v2023q4, https://david.ncifcrf.gov/) to utilize Kyoto Encyclopedia of Genes and Genomes (KEGG) database. A p-value threshold of 0.05 was used as a filter during the analysis. After pathway analysis, enrichment bubble plots were created for pathways with enrichment scores, p-values, and count values *via* the online SRplot program (available at https://www.bioinformatics.com.cn/) (Tang et al., 2023).

### Statistics

The data obtained in this study were statistically analyzed using SPSS (version 27, SPSS Inc., Chicago, United States) software. An independent samples t-test was used to determine the differences between the groups. The experimental design flowchart was generated using BioRender software (BioRender, Toronto, Canada, https://www.biorender.com/), while the behavioral graphs were created using GraphPad Prism (GraphPad Software, version 9.5.1 for Windows, Boston, Massachusetts United States, www.graphpad.com). Data are presented as mean ± S.D. values. Throughout the study, p values <0.05 were considered as statistically significant.

## Results

The present study demonstrates that chronic CRD significantly alters dentine regeneration following DPI. No mortality was observed during or after the DPI procedure. Animals maintained stable general health throughout the experimental period, with no significant differences in body weight or food and water intake between groups (*data not shown*).

### CRD alters locomotor activity and anxiety-like behavior

**FIGURE 2:**
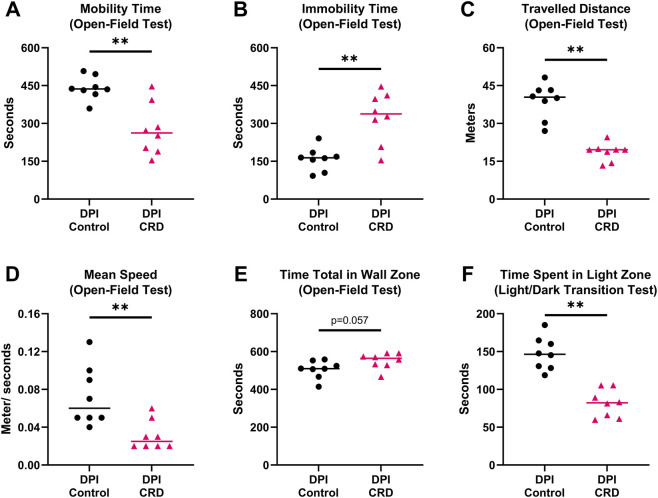
Effects of CRD on locomotor activity and anxiety-like behavior. Open-field test parameters included **(A)** mobility time, **(B)** immobility time, **(C)** traveled distance, **(D)** mean speed, and **(E)** total time spent in the wall zone. Anxiety-like behavior was further assessed using the light/dark transition test, where **(F)** time spent in the light compartment was quantified. Data are presented as mean ± SD. **p < 0.01/*p < 0.05 compared with the control group.

In the open-field test, mice exposed to CRD exhibited significantly reduced mobility time, travelled distance, and mean speed compared with control mice, accompanied by a significant increase in immobility time (p < 0.01 for all parameters; Figures 2A–D). Time spent in the wall zone showed a non-significant (p = 0.057) trend toward increase in the CRD group (Figure 2E). In the light/dark transition test, CRD mice spent significantly less time in the light area compared with controls, indicating increased anxiety-like behavior (Figure 2F).

### CRD impairs time-dependent dentine regeneration following DPI

**FIGURE 3:**
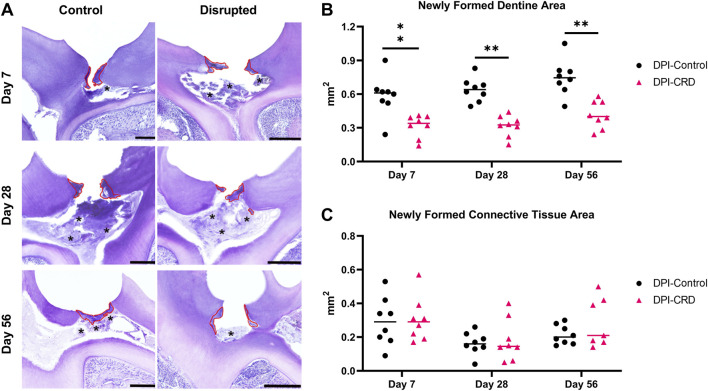
Histological and quantitative assessment of dentine and connective tissue regeneration following DPI under normal and CRD conditions. **(A)** Representative H&E-stained sections showing newly formed dentine and connective tissue within the injured pulp area (scale bars: 200 μm). **(B)** Quantification of newly formed dentine area at days 7, 28, and 56 post-injury. **(C)** Quantification of newly formed connective tissue area at the same point. Data are represented as mean +S.D. **p < 0.01 compared with the control group.

Histological evaluation revealed a characteristic reparative response following DPI, with newly formed calcified dentine surrounding the peripheral injury site and central connective tissue occupying the lesion core (Figure 3A). Complete closure of the defect was not observed in all groups; however, in control mice at day 56, the apical portion of the injury was fully sealed with regenerated dentine. Importantly, no complete loss of pulp vitality was observed in any experimental group at any time point, as evidenced by preserved pulp tissue architecture and absence of necrotic features.

Histomorphometric analysis demonstrated a progressive increase in newly formed dentine area from day 7 to day 56 in control mice, indicating a continuous reparative process (Figure 3B). Although a similar temporal pattern was observed in CRD mice, dentine regeneration occurred at a significantly reduced rate. CRD resulted in a significant reduction in regenerated dentine area at all evaluated time points (days 7, 28, and 56), indicating a consistently impaired dentinogenic response under disrupted circadian conditions. It was suggested that along with mature odontoblasts’ low activation capacity, the dentine regeneration rate is also slow after injury (Lee et al., 2023).

In contrast to dentine formation, connective tissue regeneration exhibited greater variability (Figure 3C). While connective tissue area remained relatively stable across time points in control mice, CRD mice showed a modest, non-significant tendency toward increased connective tissue formation at each time point. However, these differences did not reach statistical significance, indicating that CRD primarily affected dentine regeneration rather than connective tissue deposition.

### Identification of proteomic alterations following dental injury

To disentangle injury-driven from circadian-driven proteomic remodeling, we performed three predefined comparisons. First, Sham-Control *versus* DPI-Control was used to define injury-associated proteomic signatures under normal circadian conditions. Second, Sham-Control *versus* Sham-CRD was used to define circadian-dependent remodeling under physiological, non-injured conditions. Third, DPI-Control *versus* DPI-CRD directly assessed how CRD modifies the injury-associated proteomic response at day 56. These datasets were then integrated to resolve injury-specific, circadian-specific, shared, and context-dependent protein modules.

**FIGURE 4:**
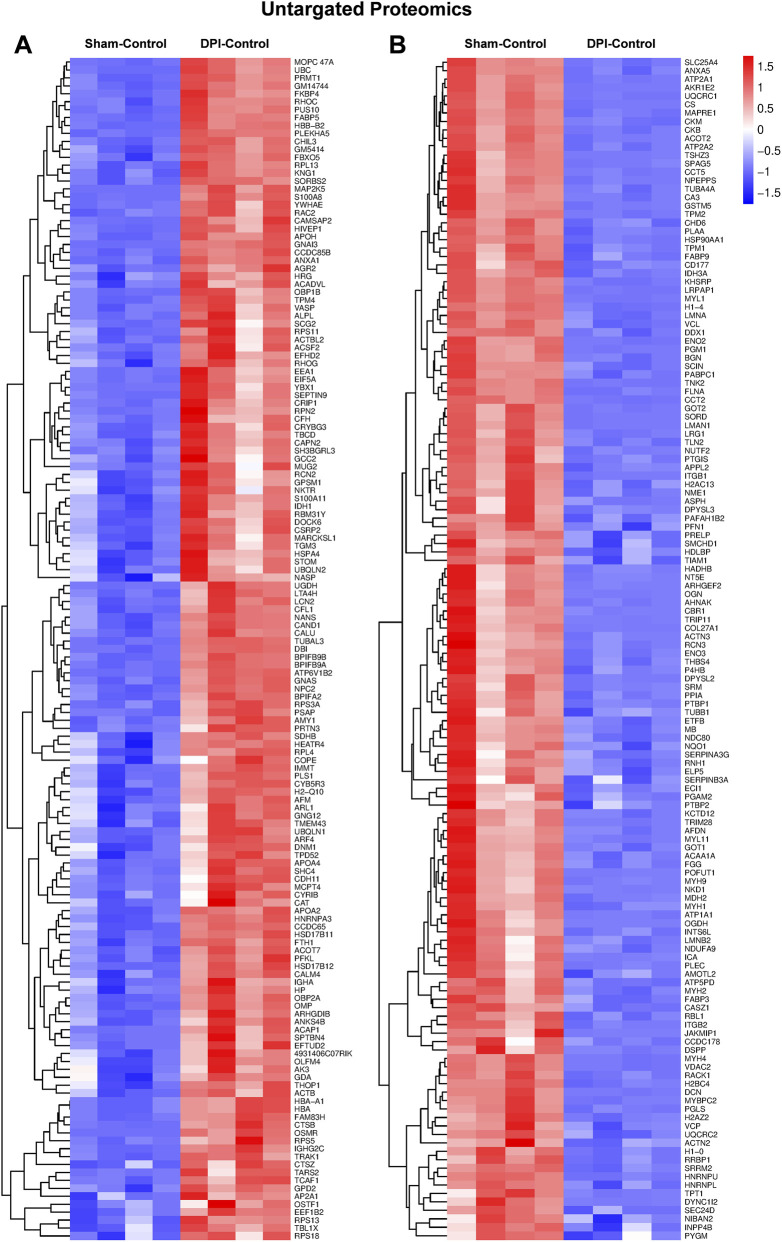
Untargeted proteomic profiling reveals injury-associated molecular remodeling under normal circadian conditions. Untargeted proteomic profiling reveals injury-associated molecular remodeling under normal circadian conditions. Heatmaps show hierarchical clustering of DEPs identified by untargeted proteomic analysis in sham and injured dental tissues (Sham-Control vs. DPI-Control). **(A)** Proteins significantly increased after DPI (p < 0.01, fold change ≥2). **(B)** Proteins significantly decreased after DPI (p < 0.01, fold change ≥2). The color scale reflects Z-score-normalized protein abundance, with red indicating higher and blue indicating lower expression.

To define injury-induced proteomic changes, untargeted LC-MS/MS analysis was performed on dental tissues obtained from Sham-Control and DPI-Control mice. A total of approximately 1,200 proteins were identified across samples. Comparative analysis revealed robust injury-associated remodeling of the dental proteome, with 150 proteins significantly upregulated and 140 proteins significantly downregulated following DPI (p < 0.01, fold change ≥2; Figures 4A,B).

**FIGURE 5:**
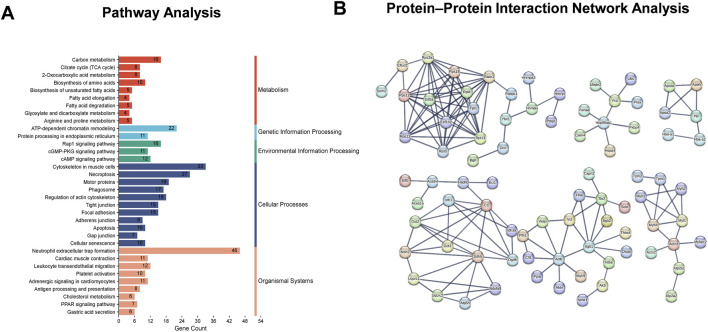
Pathway enrichment and PPI network analysis of DEPs. **(A)** KEGG pathway enrichment analysis of DEPs performed using DAVID and visualized *via* SRplot, highlighting enriched metabolic pathways and cellular process-related categories. **(B)** Protein-protein interaction (PPI) network constructed using STRING with a high-confidence interaction score (0.900), illustrating interaction patterns and network organization among DEPs.

Pathway enrichment analysis using DAVID demonstrated that differentially expressed proteins (DEPs) were predominantly associated with metabolic pathways, including carbon metabolism, the tricarboxylic acid (TCA) cycle, glycolysis/gluconeogenesis, fatty-acid degradation, and amino-acid biosynthesis (Figure 5A). In addition, enrichment was observed in cellular processes related to protein processing, cytoskeletal organization, necroptosis, and motor protein activity, consistent with the metabolic and structural demands of tissue repair. Gene Ontology (GO) analysis supported these findings, revealing enrichment of biological processes related to energy metabolism and oxygen transport (Supplementary Figure S1A). Cellular component analysis highlighted associations with nucleosome core structures, contractile filaments, and immune-related complexes, while molecular function categories were enriched for actin- and myosin-binding proteins and structural constituents, indicating coordinated cytoskeletal and translational responses to injury.

PPI network analysis identified several tightly connected modules reflecting coordinated regulation of translational machinery, mitochondrial metabolism, and extracellular matrix organization (Figure 5B). The translational module was centered around ribosomal proteins such as Large ribosomal subunit protein uL4 (RPL4) and Small ribosomal subunit protein uS13 (RPS18), while the metabolic cluster included key enzymes of the TCA cycle, including citrate synthase (CS) and succinate dehydrogenase subunit B (SDHB). In addition, an extracellular matrix-associated module featured structural and adhesion-related proteins such as integrin beta-1 (ITGB1) and dentine sialophosphoprotein (DSP). These interaction clusters indicate that dental injury triggers an integrated proteomic response involving metabolic reprogramming, cytoskeletal remodeling, and matrix-associated repair processes.

### Proteomic remodeling induced by CRD under physiological condition

**FIGURE 6:**
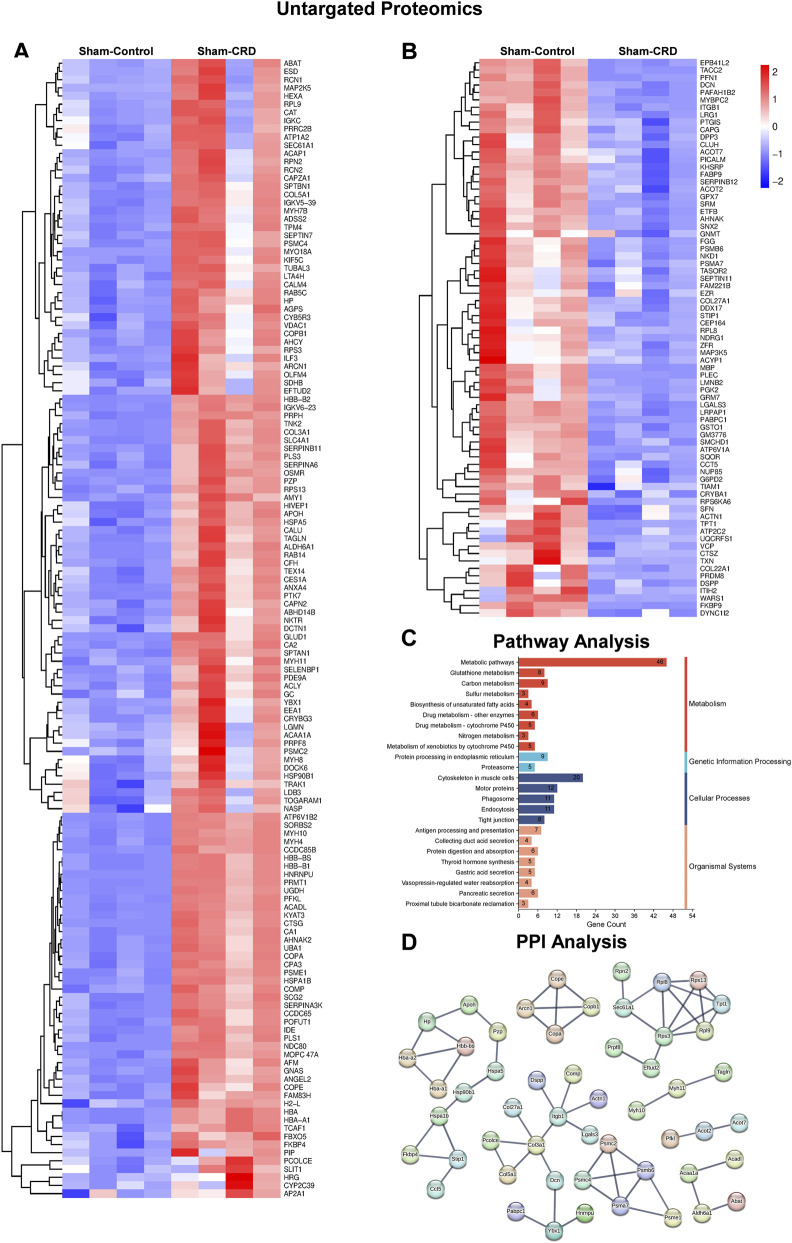
Untargeted proteomic profiling, pathway enrichment, and interaction network analysis in dental tissues under normal and CRD conditions. **(A)** Proteins significantly increased and **(B)** proteins significantly decreased in the CRD group relative to controls, showing clear group separation based on Z-score-normalized abundance patterns (Sham-Control vs. Sham-CRD). **(C)** KEGG pathway enrichment analysis of circadian-dependent DEPs, with gene counts indicating the number of proteins contributing to each enriched pathway. **(D)** PPI network of circadian-dependent DEPs constructed using STRING with a high-confidence interaction score, illustrating interaction patterns among regulated proteins.

To assess the impact of chronic CRD on the dental proteome under physiological conditions, untargeted LC-MS/MS analysis was performed on dental tissues from Sham-Control and Sham-CRD mice. Comparative analysis identified substantial circadian-dependent remodeling, with 139 proteins significantly increased and 75 proteins significantly decreased in the CRD group (p < 0.01, fold change ≥2; Figures 6A,B).

Pathway enrichment analysis revealed that CRD predominantly affected metabolic processes, including glutathione metabolism, carbon metabolism, sulfur metabolism, nitrogen metabolism, and fatty-acid metabolism (Figure 6C). In addition to these core metabolic pathways, enrichment was observed in cellular processes related to cytoskeletal organization, intracellular trafficking, endocytosis, and proteasome function, indicating broad alterations in structural and proteostatic regulation under circadian misalignment. Notably, several disease-associated pathway annotations were also enriched in a descriptive manner, reflecting the involvement of shared molecular components commonly implicated in metabolic and inflammatory disorders. These annotations were not interpreted as disease-specific mechanisms but rather highlight the translational relevance of circadian-dependent proteomic remodeling in dental tissue. GO analysis further supported these findings, showing enrichment of biological processes related to transport, oxygen transport, cell shape regulation, and lipid metabolism (Supplementary Figure S1B). Cellular component analysis highlighted associations with thick filament structures, proteasomes, kinetochores, and peroxisomes, while molecular function categories were enriched for actin- and myosin-associated activities and oxidative enzymes, consistent with coordinated metabolic and cytoskeletal remodeling (Supplementary Figure S1B).

PPI network analysis revealed several interconnected modules, indicating coordinated regulation of metabolic, structural, and proteostatic pathways under CRD (Figure 6D). Key clusters included a ribosomal and translational network centered around small ribosomal subunit protein uS15 (RPS13), 60S ribosomal protein L9 (RPL9), large ribosomal subunit protein uL2 (RPL8), large ribosomal subunit protein uL3 (RPL3) and translationally-controlled tumor protein (TPT1); an extracellular matrix and cell-adhesion network involving ITGB1, decorin (DCN), and collagen alpha-1(III) chain (COL3A1); and a metabolic cluster featuring enzymes such as SDHB and long-chain specific acyl-CoA dehydrogenase (ACADL). Additional nodes included chaperone-associated proteins (endoplasmic reticulum chaperone BiP (HSPA5), heat shock protein HSP 90-alpha (HSP90AA1) and secret or structural proteins linked to dentine and connective tissue organization (DSP, cartilage oligomeric matrix protein (COMP).

### Integrated analysis of injury- and circadian-dependent proteomic remodeling

**FIGURE 7:**
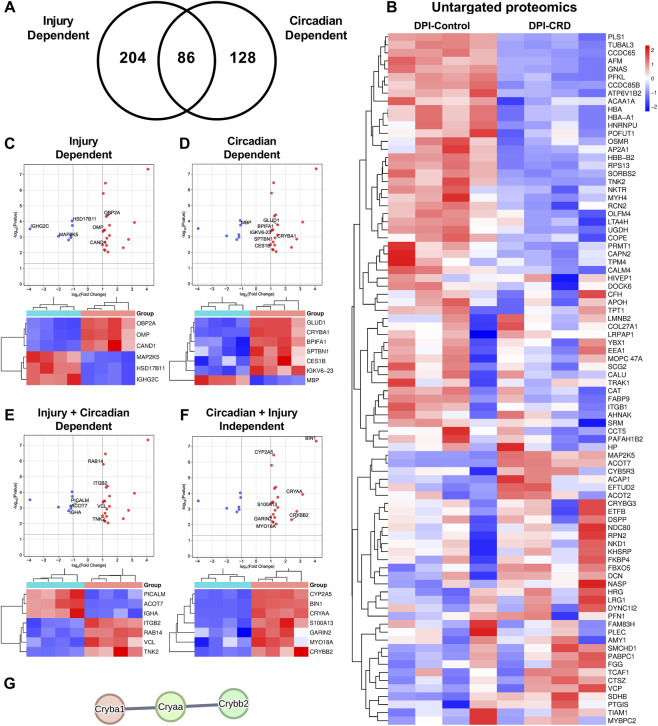
Integrated analysis of injury- and circadian-dependent proteomic remodeling in dental tissue. **(A)** Venn diagram showing the distribution of DEPs identified in the injury-dependent comparison (Sham-Control vs. DPI-Control) and the circadian-dependent comparison under physiological conditions (Sham-Control vs. Sham-CRD), highlighting 204 injury-specific proteins, 128 circadian-specific proteins, and a shared subset of 86 proteins regulated by both DPI and CRD. **(B)** Heatmap showing the expression patterns of the 86 shared proteins across experimental groups. **(C–F)** To determine how CRD modifies injury-associated proteomic programs, a direct comparison between DPI-Control and DPI-CRD groups at day 56 was performed, identifying 27 DEPs. These proteins were classified into four regulatory patterns based on their expression behavior **(C)** proteins predominantly regulated by injury, **(D)** proteins predominantly regulated by circadian disruption, **(E)** proteins jointly influenced by injury and circadian disruption, and **(F)** proteins whose expression remained largely independent of either condition. **(G)** PPI network analysis of the regulated proteins reveals a tightly connected cluster enriched for crystallin family members, indicating a conserved stress-adaptive module preserved across injury and CRD conditions.

To examine the relationship between injury- and circadian-dependent proteomic remodeling, protein sets identified in the injury-dependent (Sham-Control vs. DPI-Control; Figures 4A,B) and circadian-dependent (Sham-Control vs. Sham-CRD; Figures 6A,B) analyses were directly compared. This overlap analysis revealed 204 proteins uniquely regulated by DPI, 128 proteins uniquely altered by CRD, and a shared subset of 86 proteins that were sensitive to both injury and circadian disruption (Figures 7A,B). Thus, these 86 proteins represent a molecular intersection between dentine injury responses and circadian regulation.

To determine which of these circadian-sensitive injury-related proteins were differentially regulated under CRD conditions during injury, a direct comparison between DPI-Control and DPI-CRD groups was performed at day 56. This analysis identified 27 proteins whose expression differed significantly between DPI-Control and DPI-CRD mice (p < 0.01, ≥2-fold change), representing a subset of the shared protein pool that is specifically modulated by CRD in the context of injury. These proteins were subsequently classified into four regulatory categories based on their expression patterns (Figures 7C–F).

The injury-dependent group included proteins associated with stress response and protein turnover, including odorant-binding protein 2a (OBP2A), olfactory marker protein (OMP), and cullin-associated NEDD8-dissociated protein 1 (CAND1), which were increased following DPI. In contrast, proteins linked to growth-factor signaling and metabolic regulation, such as mitogen-activated protein kinase 5 (MAP2K5), estradiol 17-beta-dehydrogenase 11 (HSD17B11), and immunoglobulin heavy constant gamma 2C (IGHG2C), were decreased (Figure 7C).

Circadian-dependent proteins were enriched for metabolic and structural regulators, with increased expression of glutamate dehydrogenase 1 (GLUD1), Beta-A3/A1 crystallin protein (CRYBA1), BPI fold-containing family A member 1 (BPIFA1), spectrin beta chain, non-erythrocytic 1 (SPTBN1), and carboxylic ester hydrolase (CEs1B). Reduced expressions were observed for immune- and neural-associated proteins such as immunoglobulin kappa variable 6-23 (IGKV6-23) and myelin basic protein (MBP) (Figure 7D). Proteins jointly regulated by both injury and CRD showed increased expression of vinculin (VCL), activated CDC42 kinase 1 (TNK2), Ras-related protein Rab-14 (RAB14), and integrin beta-2 (ITGB2). In contrast, phosphatidylinositol-binding clathrin assembly protein (PICALM), immunoglobulin heavy constant alpha (IGHA), and cytosolic acyl coenzyme A thioester hydrolase (ACOT7) were decreased in this group (Figure 7E).

The condition-independent group was characterized by marked upregulation of stress-responsive and structural proteins, including cytochrome P450 (CYP2A5), myc box-dependent-interacting protein 1 (BIN1), alpha-crystallin A chain (CRYAA), protein S100-A13 (S100A13), family with sequence similarity 71 member D (GARIN2), myosin XVIIIA (MYO18A), and beta-crystallin B2 (CRYBB2) (Figure 7F). Consistent with these findings, PPI analysis revealed a tightly connected crystallin-centered cluster composed of CRYBA1, CRYAA, and CRYBB2, indicating a coordinated regulatory module among crystallin family members that was preserved across injury and circadian disruption conditions (Figure 7G).

## Discussion

Circadian rhythms regulate physiological homeostasis and coordinate stress responses that can influence tissue repair. In the present study, chronic CRD produced a stress-related behavioral phenotype, reflected by reduced locomotor activity and increased anxiety-like behavior (Agorastos et al., 2019; Schurhoff and Toborek, 2023). This phenotype confirms sustained circadian misalignment in the CRD group and provides a relevant systemic context for evaluating regenerative outcomes.

Under physiological conditions, DPI triggered a canonical reparative response characterized by connective tissue formation followed by peripheral mineralization and tertiary dentine deposition, consistent with established descriptions of reparative dentinogenesis (Magloire et al., 1988; Simon et al., 2011). In contrast, circadian-disrupted animals exhibited a marked attenuation of this regenerative process. Histomorphometric analyses demonstrated that both the thickness and total area of newly formed dentine were significantly reduced under CRD, indicating that intact circadian regulation is required for efficient odontoblast-like cell differentiation and subsequent mineral deposition. This finding aligns with accumulating evidence that mineralized tissues express circadian-regulated programs and that temporal misalignment compromises hard tissue formation (He et al., 2013; Pragnere et al., 2021). Collectively, these results identify circadian rhythm integrity as a critical determinant of effective dentine regeneration following pulp injury. From a translational perspective, these findings may be relevant for regenerative endodontic procedures that rely on efficient pulp healing and tertiary dentine formation.

To our knowledge, this is the first study demonstrating that both dentine injury and chronic CRD substantially reshape the dental proteome. Quantitative mass spectrometry identified approximately 1,120 proteins per condition, with DPI driving a coordinated remodeling of proteins involved in cellular metabolism, cytoskeletal organization, immune responses, and extracellular matrix dynamics. This broad proteomic shift reflects the complex bioenergetic, structural, and inflammatory requirements of early reparative dentinogenesis. PPI analysis revealed three dominant functional modules activated following injury: a translation-associated cluster centered on ribosomal proteins (RPL4, RPS18, RPL13), a metabolic cluster comprising key tricarboxylic acid cycle enzymes (CS, SDHB, OGDH), and a structural remodeling module enriched in cytoskeletal and extracellular matrix-related proteins (ITGB1, ACTB, THBS4, DSP). Together, these modules illustrate the integrated engagement of biosynthetic capacity, energy metabolism, and structural reorganization during the initiation of dentine repair.

Beyond injury-driven changes, chronic CRD independently altered the basal dental proteome, indicating that circadian misalignment profoundly influences protein expression in dental tissue. Importantly, our integrated analysis separated circadian effects on basal tissue homeostasis from circadian modulation of the injury response. Across the injury-dependent (Sham-Control vs. DPI-Control) and circadian-dependent (Sham-Control vs. Sham-CRD) comparisons, we identified an intersecting set of 86 proteins that were sensitive to both dental injury and circadian disruption, representing a molecular interface between regenerative programs and circadian regulation. However, only a smaller subset of 27 proteins differed directly between DPI-Control and DPI-CRD mice at day 56, indicating that circadian misalignment does not uniformly shift the entire injury signature but selectively reprograms specific components of the injury-associated proteomic response. This distinction supports the concept that CRD modifies dentine repair through targeted perturbation of metabolic, cytoskeletal, and immune-trafficking pathways that are recruited during regeneration rather than by globally amplifying or suppressing injury-driven remodeling.

The circadian-dependent alterations predominantly involved metabolic enzymes, components of the translational machinery, and extracellular matrix-associated proteins, emphasizing the role of circadian timing in maintaining dental tissue homeostasis. Network-level organization highlighted coordinated changes in proteostatic and stress-adaptive programs, reflected by altered expression of molecular chaperones (HSPA5, HSP90AA1) and key extracellular matrix components (DCN, COL3A1, COMP), consistent with a proteomic environment less permissive for efficient dentine regeneration.

Stratification of differentially expressed proteins into injury-dependent and circadian-dependent groups provided insight into how chronic CRD reshapes the molecular response to dentine injury. Injury-dependent alterations were primarily associated with stress adaptation, proteostasis, and regulatory signaling. Increased expression of OBP2A and OMP, previously linked to epithelial and neuronal stress contexts, is consistent with cellular adaptation to pulp injury (Dibattista and Reisert, 2016; Jeong et al., 2023). Upregulation of CAND1 further supports enhanced ubiquitin-dependent protein turnover during tissue remodeling (Werner et al., 2017; Zhang et al., 2023). In contrast, reduced levels of MAP2K5 and HSD17B11 reflect modulation of growth factor-related signaling and metabolic regulation following injury (Huang et al., 2021).

CRD alone affected a distinct subset of metabolic and structural regulators, reflecting a basal proteomic state shaped by chronic temporal misalignment. Altered expression of GLUD1, a mitochondrial glutamate dehydrogenase involved in amino acid-driven energy production, is consistent with circadian-dependent rewiring of mitochondrial metabolism under CRD (Lu et al., 2019). Given the high energetic demands of pulp repair and mineralization, such metabolic shifts may reduce the cellular capacity to support regenerative processes. In parallel, increased expression of BPIFA1, an epithelial defense and stress-response protein (Chua et al., 2011; Wang et al., 2021), together with CEs1B, a regulator of lipid and xenobiotic metabolism (Lian et al., 2018), suggests activation of protective and detoxification programs associated with epithelial and metabolic stress. Changes in SPTBN1, a spectrin family scaffolding protein that links cytoskeletal organization to membrane stability and TGF-β signaling, indicate increased vulnerability of the cytoskeletal framework under CRD. Concurrent reductions in immune- and neural-associated proteins, including IGKV6-23, which reflects local immunoglobulin repertoire dynamics (Barbie and Lefranc, 1998), and MBP, a key structural component associated with neural integrity (Nevinsky et al., 2022), further point to impaired neuroimmune homeostasis.

Proteins jointly regulated by both injury and circadian disruption-particularly those directly altered between DPI-Control and DPI-CRD-highlight a mechanistic convergence between tissue damage and circadian control. Key nodes within this shared axis included mechanosensitive and trafficking-related proteins such as VCL, TNK2/ACK1, and RAB14, underscoring the importance of adhesion dynamics, cytoskeletal organization, and vesicular transport during repair (Holle et al., 2013; Lu et al., 2014; Mahajan and Mahajan, 2015). Modulation of immune and endocytic regulators, including ITGB2 and PICALM, further indicates that circadian misalignment alters the cellular infrastructure required for coordinated inflammatory and reparative responses.

A subset of proteins exhibited consistent regulation regardless of injury or circadian state, representing a conserved stress-adaptive module. Prominent among these were crystallins (CRYAA, CRYAB, CRYBB2), which formed a tightly connected interaction cluster and are known to function as molecular chaperones protecting cells against oxidative and proteotoxic stress (Yu et al., 2012; Yu et al., 2021). The concurrent involvement of additional stress-responsive and cytoskeletal proteins, including S100A13, BIN1, and MYO18A, is consistent with activation of cytoprotective and structural stabilization mechanisms under combined mechanical injury and circadian disruption.

Importantly, the present findings extend beyond experimental circadian biology and may have direct implications for vital pulp therapy and regenerative endodontics. Although this study was conducted in a murine model, the observed reduction in tertiary dentine formation and the circadian-dependent remodeling of regenerative pathways suggest that circadian rhythm integrity may represent a previously underrecognized biological factor influencing reparative outcomes after pulp injury. From a translational perspective, these findings support the concept that systemic chronobiological status may influence the biological response to pulp injury and repair. These observations further raise the possibility that chronobiology-informed therapeutic strategies or optimization of treatment timing may improve regenerative predictability in future dental applications. Nevertheless, future translational and clinical studies are required to determine whether circadian disruption similarly affects healing outcomes in human dental tissues.

Several limitations of this study should be acknowledged. The experiments were conducted in a murine model, which allows controlled investigation of pulp injury and regeneration but may limit direct translation to human clinical settings due to interspecies differences. In addition, proteomic analyses were performed at a single post-injury time point, which does not capture time-dependent changes in protein expression during the dynamic phases of dentine repair. Moreover, the study focused on proteomic profiling, and integration with complementary multi-omics approaches, such as transcriptomics or metabolomics, was beyond the scope of the present work but may provide further mechanistic insight.

## Conclusion

Chronic CRD impaired tertiary dentine formation following DPI in mice. Proteomic analyses further demonstrated that circadian misalignment alters molecular pathways associated with tissue repair and regeneration. These findings identify circadian rhythm integrity as a potential biological factor influencing pulp healing and suggest that chronobiology may have translational relevance for vital pulp therapy and regenerative endodontics.

## Data Availability

The mass spectrometry proteomics data generated in this study have been deposited in the jPOST repository and are available through the ProteomeXchange Consortium under accession number PXD079588 (jPOST accession: JPST004688).

## References

[B1] Agorastos A. Nicolaides N. C. Bozikas V. P. Chrousos G. P. Pervanidou P. (2019). Multilevel interactions of stress and circadian system: implications for traumatic stress. Front. Psychiatry 10, 1003. 10.3389/fpsyt.2019.01003 32047446 PMC6997541

[B2] Barbie V. Lefranc M. P. (1998). The human immunoglobulin kappa variable (IGKV) genes and joining (IGKJ) segments. Exp. Clin. Immunogenet 15 (3), 171–183. 10.1159/000019068 9813414

[B3] Beker M. C. Caglayan B. Yalcin E. Caglayan A. B. Turkseven S. Gurel B. (2018). Time-of-Day dependent neuronal injury after ischemic stroke: implication of circadian clock transcriptional factor Bmal1 and survival kinase AKT. Mol. Neurobiol. 55 (3), 2565–2576. 10.1007/s12035-017-0524-4 28421530

[B4] Beker M. C. Pence M. E. Yagmur S. Caglayan B. Caglayan A. Kilic U. (2022). Phosphodiesterase 10A deactivation induces long-term neurological recovery, Peri-infarct remodeling and pyramidal tract plasticity after transient focal cerebral ischemia in mice. Exp. Neurol. 358, 114221. 10.1016/j.expneurol.2022.114221 36075453

[B5] Beker M. C. Aydinli F. I. Caglayan A. B. Beker M. Baygul O. Caglayan A. (2023). Age-associated resilience against ischemic injury in mice exposed to transient middle cerebral artery occlusion. Mol. Neurobiol. 60 (8), 4359–4372. 10.1007/s12035-023-03353-4 37093494

[B6] Brizuela C. Ormeno A. Cabrera C. Cabezas R. Silva C. I. Ramirez V. (2017). Direct pulp capping with calcium hydroxide, mineral trioxide aggregate, and biodentine in permanent young teeth with caries: a randomized clinical trial. J. Endod. 43 (11), 1776–1780. 10.1016/j.joen.2017.06.031 28917577

[B7] Chua Y. S. Boh B. K. Ponyeam W. Hagen T. (2011). Regulation of cullin RING E3 ubiquitin ligases by CAND1 *in vivo* . PLoS One 6 (1), e16071. 10.1371/journal.pone.0016071 21249194 PMC3020946

[B8] Coll J. A. Dhar V. Guelmann M. Crystal Y. O. Chen C. Y. Marghalani A. A. (2025a). Guideline for use of vital pulp therapy in permanent teeth. Pediatr. Dent. 47 (5), 299–311. 41121563

[B9] Coll J. A. Dhar V. Guelmann M. Crystal Y. O. Chen C. Y. Marghalani A. A. (2025b). Vital pulp therapy in permanent teeth: a systematic review and meta-analyses. Pediatr. Dent. 47 (3), 137–150. 40533920

[B10] Dibattista M. Reisert J. (2016). The odorant receptor-dependent role of olfactory marker protein in olfactory receptor neurons. J. Neurosci. 36 (10), 2995–3006. 10.1523/JNEUROSCI.4209-15.2016 26961953 PMC4783500

[B11] Duncan H. F. Galler K. M. Tomson P. L. Simon S. El-Karim I. Kundzina R. (2019). European society of endodontology position statement: management of deep caries and the exposed pulp. Int. Endod. J. 52 (7), 923–934. 10.1111/iej.13080 30664240

[B12] He Y. Chen Y. Zhao Q. Tan Z. (2013). Roles of brain and muscle ARNT-like 1 and Wnt antagonist Dkk1 during osteogenesis of bone marrow stromal cells. Cell. Prolif. 46 (6), 644–653. 10.1111/cpr.12075 24460718 PMC6495916

[B13] Holle A. W. Tang X. Vijayraghavan D. Vincent L. G. Fuhrmann A. Choi Y. S. (2013). *In situ* mechanotransduction *via* vinculin regulates stem cell differentiation. Stem Cells 31 (11), 2467–2477. 10.1002/stem.1490 23897765 PMC3833960

[B14] Huang Y. Wang P. Morales R. Luo Q. Ma J. (2021). Map2k5-Deficient mice manifest phenotypes and pathological changes of dopamine deficiency in the central nervous system. Front. Aging Neurosci. 13, 651638. 10.3389/fnagi.2021.651638 34168549 PMC8217467

[B15] Iwamoto C. E. Adachi E. Pameijer C. H. Barnes D. Romberg E. E. Jefferies S. (2006). Clinical and histological evaluation of white ProRoot MTA in direct pulp capping. Am. J. Dent. 19 (2), 85–90. 16764130

[B16] Janjic K. Agis H. (2019). Chronodentistry: the role and potential of molecular clocks in oral medicine. BMC Oral Health 19 (1), 32. 10.1186/s12903-019-0720-x 30760278 PMC6375164

[B17] Jeong J. H. Zhong S. Li F. Huang C. Chen X. Liu Q. (2023). Tumor-derived OBP2A promotes prostate cancer castration resistance. J. Exp. Med. 220 (3), e20211546. 10.1084/jem.20211546 36547668 PMC9789742

[B18] Kosin K. Liszka W. Polak A. Malina M. Kiwior J. (2025). Modern biomaterials for direct pulp capping: a literature review. Wiad. Lek. 78 (9), 1893–1898. 10.36740/WLek/209474 41160870

[B19] Lawther A. J. Phillips A. J. Chung N.-C. Chang A. Ziegler A. I. Debs S. (2022). Disrupting circadian rhythms promotes cancer-induced inflammation in mice. Brain, Behav. and Immunity-Health 21, 100428. 10.1016/j.bbih.2022.100428 35199050 PMC8851215

[B20] Lee M. Lee Y. S. Shon W. J. Park J. C. (2023). Physiologic dentin regeneration: its past, present, and future perspectives. Front. Physiol. 14, 1313927. 10.3389/fphys.2023.1313927 38148896 PMC10750396

[B21] Li Z. Cao L. Fan M. Xu Q. (2015). Direct pulp capping with calcium hydroxide or mineral trioxide aggregate: a meta-analysis. J. Endod. 41 (9), 1412–1417. 10.1016/j.joen.2015.04.012 25990198

[B22] Li X. L. Fan W. Fan B. (2024). Dental pulp regeneration strategies: a review of status quo and recent advances. Bioact. Mater. 38, 258–275. 10.1016/j.bioactmat.2024.04.031 38745589 PMC11090883

[B23] Lian J. Nelson R. Lehner R. (2018). Carboxylesterases in lipid metabolism: from mouse to human. Protein Cell. 9 (2), 178–195. 10.1007/s13238-017-0437-z 28677105 PMC5818367

[B24] Lu R. Johnson D. L. Stewart L. Waite K. Elliott D. Wilson J. M. (2014). Rab14 regulation of claudin-2 trafficking modulates epithelial permeability and lumen morphogenesis. Mol. Biol. Cell. 25 (11), 1744–1754. 10.1091/mbc.E13-12-0724 24694596 PMC4038501

[B25] Lu X. Liu S. F. Wang H. H. Yu F. Liu J. J. Zhao Y. M. (2019). A biological study of supernumerary teeth derived dental pulp stem cells based on RNA-seq analysis. Int. Endod. J. 52 (6), 819–828. 10.1111/iej.13060 30565714

[B26] Magloire H. Joffre A. Hartmann D. J. (1988). Localization and synthesis of type III collagen and fibronectin in human reparative dentine. Immunoperoxidase and immunogold staining. Histochemistry 88 (2), 141–149. 10.1007/BF00493296 3279013

[B27] Mahajan K. Mahajan N. P. (2015). ACK1/TNK2 tyrosine kinase: molecular signaling and evolving role in cancers. Oncogene 34 (32), 4162–4167. 10.1038/onc.2014.350 25347744 PMC4411206

[B28] Mendoza J. (2025). Brain circadian clocks timing the 24h rhythms of behavior. Npj Biol. Timing Sleep 2 (1), 13. 10.1038/s44323-025-00030-8 41776262 PMC12912386

[B29] Morotomi T. Washio A. Kitamura C. (2019). Current and future options for dental pulp therapy. Jpn. Dent. Sci. Rev. 55 (1), 5–11. 10.1016/j.jdsr.2018.09.001 30733839 PMC6354285

[B30] Nakashima M. Iohara K. Bottino M. C. Fouad A. F. Nor J. E. Huang G. T. (2019). Animal models for stem cell-based pulp regeneration: foundation for human clinical applications. Tissue Eng. Part B Rev. 25 (2), 100–113. 10.1089/ten.TEB.2018.0194 30284967 PMC6486672

[B31] Nevinsky G. A. Buneva V. N. Dmitrienok P. S. (2022). Multiple sclerosis: enzymatic cross site-specific recognition and hydrolysis of H2A histone by IgGs against H2A, H1, H2B, H3 histones, myelin basic protein, and DNA. Biomedicines 10 (8), 1876. 10.3390/biomedicines10081876 36009424 PMC9405453

[B32] Ohtsuka-Isoya M. Hayashi H. Shinoda H. (2001). Effect of suprachiasmatic nucleus lesion on circadian dentin increment in rats. Am. J. Physiol. Regul. Integr. Comp. Physiol. 280 (5), R1364–R1370. 10.1152/ajpregu.2001.280.5.R1364 11294755

[B33] Papakyrikos A. M. Arora M. Austin C. Boughner J. C. Capellini T. D. Dingwall H. L. (2020). Biological clocks and incremental growth line formation in dentine. J. Anat. 237 (2), 367–378. 10.1111/joa.13198 32266720 PMC7369199

[B34] Parirokh M. Torabinejad M. Dummer P. M. H. (2018). Mineral trioxide aggregate and other bioactive endodontic cements: an updated overview - part I: vital pulp therapy. Int. Endod. J. 51 (2), 177–205. 10.1111/iej.12841 28836288

[B35] Patke A. Young M. W. Axelrod S. (2020). Molecular mechanisms and physiological importance of circadian rhythms. Nat. Reviews Mol. Cell Biology 21 (2), 67–84. 10.1038/s41580-019-0179-2 31768006

[B36] Pragnere S. Auregan J. C. Bosser C. Linglart A. Bensidhoum M. Hoc T. (2021). Human dentin characteristics of patients with osteogenesis imperfecta: insights into collagen-based biomaterials. Acta Biomater. 119, 259–267. 10.1016/j.actbio.2020.10.033 33122145

[B37] Reinke H. Asher G. (2019). Crosstalk between metabolism and circadian clocks. Nat. Rev. Mol. Cell. Biol. 20 (4), 227–241. 10.1038/s41580-018-0096-9 30635659

[B38] Schurhoff N. Toborek M. (2023). Circadian rhythms in the blood-brain barrier: impact on neurological disorders and stress responses. Mol. Brain 16 (1), 5. 10.1186/s13041-023-00997-0 36635730 PMC9835375

[B39] Simon S. R. Berdal A. Cooper P. R. Lumley P. J. Tomson P. L. Smith A. J. (2011). Dentin-pulp complex regeneration: from lab to clinic. Adv. Dent. Res. 23 (3), 340–345. 10.1177/0022034511405327 21677089

[B40] Tang D. Chen M. Huang X. Zhang G. Zeng L. Zhang G. (2023). SRplot: a free online platform for data visualization and graphing. PLoS One 18 (11), e0294236. 10.1371/journal.pone.0294236 37943830 PMC10635526

[B41] Tao J. Zhai Y. Park H. Han J. Dong J. Xie M. (2016). Circadian rhythm regulates development of enamel in mouse mandibular first molar. PLoS One 11 (8), e0159946. 10.1371/journal.pone.0159946 27494172 PMC4975438

[B42] Wang L. Ren W. Li X. Zhang X. Tian H. Bhattarai J. P. (2021). Chitinase-like protein Ym2 (Chil4) regulates regeneration of the olfactory epithelium *via* interaction with inflammation. J. Neurosci. 41 (26), 5620–5637. 10.1523/JNEUROSCI.1601-20.2021 34016714 PMC8244980

[B43] Werner A. Manford A. G. Rape M. (2017). Ubiquitin-dependent regulation of stem cell biology. Trends Cell. Biol. 27 (8), 568–579. 10.1016/j.tcb.2017.04.002 28528988 PMC5643009

[B44] Yu Y. Li J. Xu J. Wang Q. Yao K. (2012). Congenital polymorphic cataract associated with a G to A splice site mutation in the human beta-crystallin gene CRYβA3/A1. Mol. Vis. 18, 2213–2220. 22919269 PMC3425576

[B45] Yu H. Liu K. Lu P. (2021). Polymorphisms in. Semin. Ophthalmol. 36 (5-6), 429–436. 10.1080/08820538.2021.1903943 34010109

[B46] Zhang X. Liu Y. Zhang T. Tan Y. Dai X. Yang Y. G. (2023). Advances in the potential roles of Cullin-RING ligases in regulating autoimmune diseases. Front. Immunol. 14, 1125224. 10.3389/fimmu.2023.1125224 37006236 PMC10064048

