# Supplementary figures and images for: Circadian rhythm disruption impairs time-dependent dentine regeneration and reprograms the late-stage dental proteome in a mouse pulp injury model
Source: Front Pharmacol. 2026 Jun 17;17:1770288. doi: 10.3389/fphar.2026.1770288 (PMC13321973; doi:10.3389/fphar.2026.1770288)

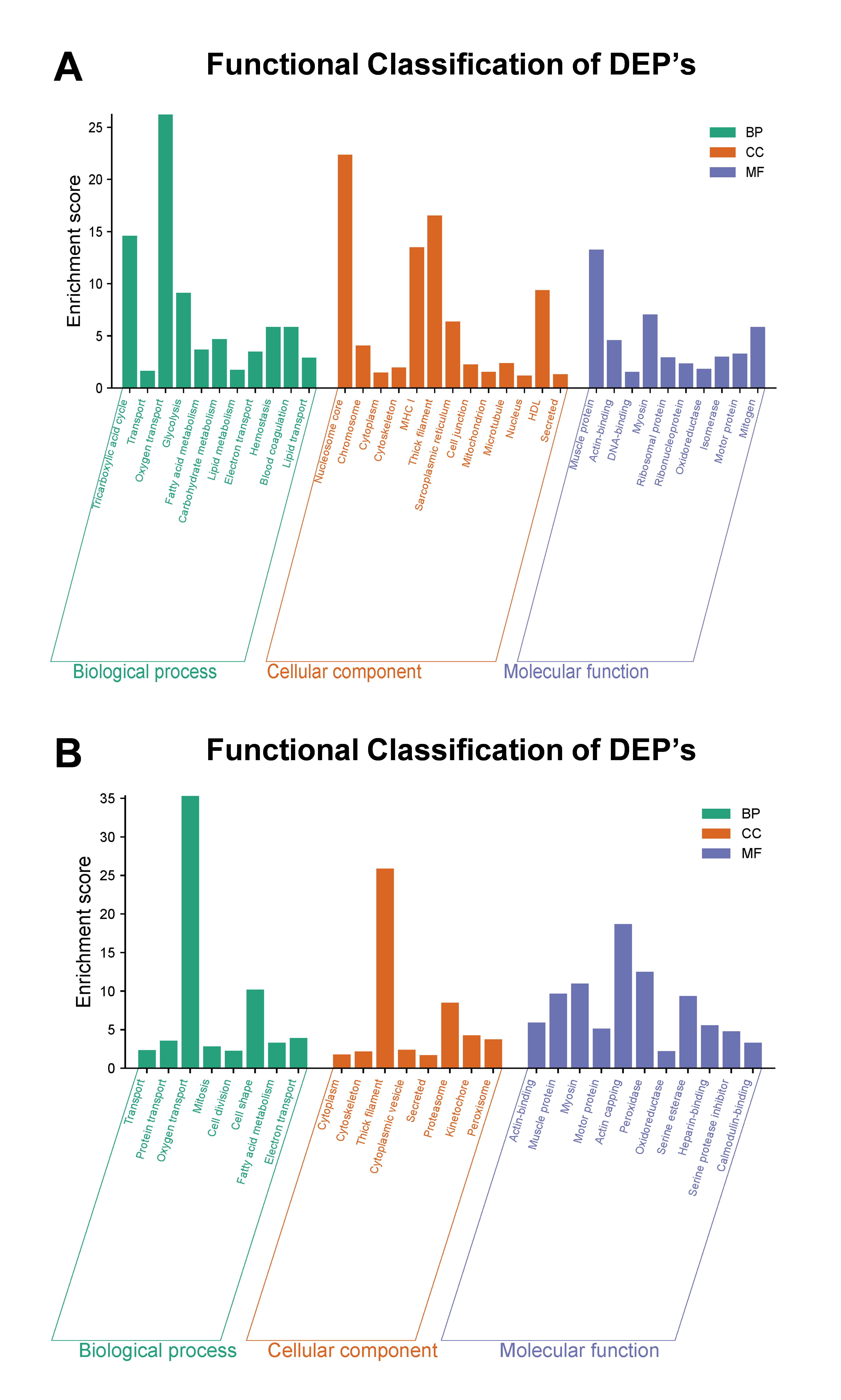

Supplement: Supplementary file 1 [file Image1.jpeg]
